# Supplementary material for: The Combined Assessment of CTC and ESR1 Status in Liquid Biopsy Samples Enhances the Clinical Value of Prediction in Metastatic Breast Cancer
Source: Int J Mol Sci. 2025 Feb 26;26(5):2038. doi: 10.3390/ijms26052038 (PMC11900918; doi:10.3390/ijms26052038)
Supplement: Supplementary file 1 [file ijms-26-02038-s001.zip › ijms-3476521-supplementary.pdf]

Supplementary table 1. The Spearman correlation analsysis for identified *ESR1* muations, *PIK3CA* mutations, and CTCs presence

Correlation matrix

	PIK3CA	***ESR1***	**CTC**
PIK3CA		0,0021	-0,1176
ESR1	0,0021		-0,0844
**CTC**	-0,1176	-0,0844	

Confidence intervals

	PIK3CA	***ESR1***	**CTC**
PIK3CA		-0,2031 to 0,2072	-0,3152 to 0,08973
ESR1	-0,2031 to 0,2072		-0,2847 to 0,1229
**CTC**	-0,3152 to 0,08973	-0,2847 to 0,1229	

p-values

	PIK3CA	***ESR1***	**CTC**
PIK3CA		0,9834	0,2513
ESR1	0,9834		0,4111
**CTC**	0,2513	0,4111	
